# A novel post-processing scheme for two-dimensional electrical impedance tomography based on artificial neural networks

**DOI:** 10.1371/journal.pone.0188993

**Published:** 2017-12-05

**Authors:** Sébastien Martin, Charles T. M. Choi

**Affiliations:** 1 Department of Electrical and Computer Engineering, National Chiao Tung University, Hsinchu, Taiwan; 2 Institute of Biomedical Engineering, National Chiao Tung University, Hsinchu, Taiwan; University of Minnesota, UNITED STATES

## Abstract

**Objective:**

Electrical Impedance Tomography (EIT) is a powerful non-invasive technique for imaging applications. The goal is to estimate the electrical properties of living tissues by measuring the potential at the boundary of the domain. Being safe with respect to patient health, non-invasive, and having no known hazards, EIT is an attractive and promising technology. However, it suffers from a particular technical difficulty, which consists of solving a nonlinear inverse problem in real time. Several nonlinear approaches have been proposed as a replacement for the linear solver, but in practice very few are capable of stable, high-quality, and real-time EIT imaging because of their very low robustness to errors and inaccurate modeling, or because they require considerable computational effort.

**Methods:**

In this paper, a post-processing technique based on an artificial neural network (ANN) is proposed to obtain a nonlinear solution to the inverse problem, starting from a linear solution. While common reconstruction methods based on ANNs estimate the solution directly from the measured data, the method proposed here enhances the solution obtained from a linear solver.

**Conclusion:**

Applying a linear reconstruction algorithm before applying an ANN reduces the effects of noise and modeling errors. Hence, this approach significantly reduces the error associated with solving 2D inverse problems using machine-learning-based algorithms.

**Significance:**

This work presents radical enhancements in the stability of nonlinear methods for biomedical EIT applications.

## Introduction

Electrical Impedance Tomography (EIT) is a method for biomedical imaging applications, and a very promising technology under active researches. In short, by applying an electrical current at the boundaries of a volume conductor (for instance, on the skin), it is possible to obtain the internal distribution of electrical conductivities by measuring the electrical voltages at the boundary of this volume and solving an inverse problem [1]. In biomedical applications, the conductivity distribution may differ according to the tissue type. Furthermore, there should be rough boundaries between two different tissues, and an accurate solution can only be obtained with a nonlinear method or with significant *a priori* knowledge of the system, which is usually not available in practice.

However, using current methods, EIT imaging reconstruction suffers from low spatial resolutions introduced by the reconstruction algorithm and the underlying assumptions usually made in order to get higher stability. The main difficulty in EIT is to obtain high-quality images, high robustness to noise, and strong resistance to modeling errors. In fact, the inverse problem is not only nonlinear, but also severely ill-posed [2]. Due to such constraints, it is usually solved by making strong assumptions about the distribution from a prior probability function [3], and using linear inverse solvers [4]. Although the solution depends on an initial estimate of the conductivity distribution, potentially causing distortions in the resultant image, linear algorithms associated with a prior probability function offer strong robustness to noise and to modeling errors such as electrode displacement.

### EIT inverse problem

To solve the nonlinear inverse problem, various algorithms have been proposed [5]. As it is technically impossible to find an explicit expression of the conductivity from a boundary measurement [6], the nonlinear problem is commonly approximated by assuming a linear solution. A summary of some commonly used methods is given below.

Linear approximations can produce satisfactory results with great robustness to noise, but the underlying reconstruction algorithm is strongly dependent on *a priori* information, and assumes that the electrical conductivity of the body varies so little from the initial estimate that this variation can be considered as linear [7]. Although the objects typically studied by finite element (FE) modeling, such as biological tissues, generally have rough boundaries, this method usually gives smooth boundaries in practice, resulting in a very crude estimate of the actual shape of the target object [8].

In contrast, nonlinear iterative methods consider the ill-posedness and nonlinearity of EIT inverse problems and use modern computational techniques that have been proved to optimize nonlinear problems. While these algorithms can provide better approximations than linear methods without substantial *a priori* knowledge of the solution, they are more sensitive to errors and usually more time-consuming. Therefore, nonlinear iterative methods are not applicable for real-time EIT [9].

Early work by Calderón [10] laid the foundation for solutions based on complex geometrical optics, later developed by Uhlmann and Sylvester [11]. The concept is based on a solution of Calderón’s inverse conductivity problem, and involves the solution of a Beltrami equation in the plane with an exponential asymptotic condition [12]. These methods have subsequently been applied for imaging cardiac activity [13].

Methods based on AI, such as ANNs, have also been proposed [14], where algorithms learn to solve the EIT inverse problem by a process known as supervised learning. After a suitable number of training iterations, they can determine the conductivity distributions directly from the measurements. AI-based methods can give a satisfactory estimate of the conductivity distribution in a short time, and are therefore suitable for real-time monitoring. It has been shown that when trained on data containing noise and reproducing other artifacts present in biomedical EIT measurements, these methods can perform accurate reconstructions from real biomedical data.

### Artificial neural networks

Artificial neural networks (ANNs) are computational models, inspired by central nervous systems, that are capable of machine learning. An ANN can be represented as a system of interconnected neurons that compute outputs from inputs when information is fed through the network [15]. ANNs can be used to implement a set of powerful algorithms to approximate the optimal solution to a nonlinear ill-posed problem. Preliminary studies have reported the use of nonlinear algorithms via ANNs [14] to replace linear or iterative inverse solvers, such as one-step Gauss—Newton (GN) [1] or the primal-dual interior point method (PDIPM) [9]. However, these solutions, based on a biomimetic approach, are usually highly sensitive to distortions and perform poorly in practical EIT reconstruction, in which the measured data usually differ from the training data and contain noise, and the electrode position is only approximate due to inevitable movements of the thorax.

### Proposed method

In this paper, a novel method is proposed to enhance the quality of EIT image reconstruction. The proposed method combines both a linear inverse solver and nonlinear algorithms. After reconstructing an EIT image using a linear EIT inverse solver, the resulting image is sent to an ANN to eliminate the smoothness and other deformations induced by the prior use of a linear method, and to recreate the rough boundaries present in the original conductivity distribution. By post-processing the EIT image obtained from the linear inverse solver, it is possible to obtain a nonlinear distribution similar to the original image. The effectiveness of this method is measured with respect to various definitions of error, and the resulting images are compared with those from a linear solver (one-step GN), a nonlinear iterative method (PDIPM), and an ANN used as a complete replacement for an EIT inverse solver. Finally, the proposed reconstruction method is applied to real medical data from the lungs. The measurements and image reconstruction are performed in two dimensions.

## Theory

### Electrical impedance tomography

In EIT, the main difficulty arises from the rough boundaries present in the conductivity distribution, for example at the boundary between two different organs. In practice, the conductivity distribution is usually considered as a linear variation from an initial estimate by the underlying mathematics of the inverse solver. The EIT problem is typically divided into two sub problems, respectively termed the forward and inverse problems.

#### The forward problem

In simulations, one has to start by solving the forward problem. This is a linear problem and can be solved numerically.

According to Maxwell’s work on electromagnetism, the electrical potential *u* in a body Ω is given by (1) [16],

∇.γ(x,ω)∇u=0 (1)

where for each point *x* in Ω and for each angular frequency *ω*, the electrical admittivity is given by (2):

γ(x,ω)=σ(x,ω)+i*ε(x,ω) (2)

where σ is the electrical conductivity and ε is the electrical permittivity. At the boundary of the domain, the mapping of the electrical voltages and electrical currents strictly depends on the conductivity of the internal body. One obtains the following boundary condition on ∂Ω (3):

$$\frac{1}{\rho }\frac{\partial V_{p}}{\partial n}\;=\;J_{I}$$ (3)

where *J*_*I*_ is the current density at the boundary ∂Ω and n represents the unit outward normal vector at the boundary. δV_ρ_ is uniquely determined by the resistivity ρ and the boundary current density J_I_ induced by I.

#### The inverse problem

Given the measured voltages at the boundary, the inverse problem estimates the internal conductivity distribution.

In this paper, the approach from Adler and Guardo [7], also known as the one-step GN method, is used to solve the inverse problem. This approach is based on a maximum *a posteriori* (MAP) method to linearize image reconstruction. A complete mathematical description of the MAP approach can be found in [17]. This method can be seen as a simplified version of the iterative GN method, where only the first step of the nonlinear method is calculated. This solution gives satisfactory reconstructions in rapid time by exploiting that in difference EIT, some parameters of the complete nonlinear method have very poor identifiability and can be set as constants [18].

Solving an inverse problem with the one-step GN method can be done by a simple matrix product. By multiplying the set of measured voltages by a reconstruction matrix, one can obtain an estimate of the conductivity distribution within the FE model. Although computing the reconstruction matrix may require time and large computing resources, it can be done prior to collecting EIT data and therefore this method can be considered as a real-time method. The reconstruction matrix is calculated according to (4), where J is the Jacobian matrix, ω the inverse covariance matrix, α an hyper parameter, and P the prior probability function [19].

$$RM\;=\;{\left(J^{T}\omega J+\alpha *P\right)}^{-1}\;*\;J^{T}\omega$$ (4)

In addition to the one-step GN method, the proposed method is also compared to iterative PDIPM and ANN-based nonlinear methods. The iterative PDIPM is based on an interior point method, also known as the barrier method. The central idea of the barrier method is to introduce a barrier function, which penalizes points that are close to the boundary of the feasible set, obviating the need for the inequality constraints [20]. In this algorithm, the applied penalty is logarithmic. As it is difficult to minimize the logarithmic barrier function, two optimality conditions must be defined, referred to as primal and dual feasibility. A complete description of the PDIPM inverse solver and its implementation for the EIT inverse problem can be found in [9].

Machine-learning-based reconstruction algorithms, such as radial basis ANNs, have been used to solve the EIT inverse problem, and show both high accuracy and rapid image reconstruction [21]. These algorithms are capable of learning to solve the inverse problem from the measured voltages. After learning, these algorithms can estimate the conductivity distribution within a relatively short time. These methods have been widely used in simulations and phantom reconstructions [22,23]. For phantom reconstructions, ANNs can perform accurately as long as the noise typically present in measured data has been considered in the training phase [24]. In real biomedical applications, the mismatch between the boundary of the FE model and the actual volume conductor should also be considered during the training phase to obtain the maximal benefits of machine-learning-based inverse solvers.

For monitoring the human breathing cycle with EIT, time-difference EIT is used. Time-difference EIT requires two measurement acquired at two different times, and obtains the image from the voltage difference between the two measurement. The resulting image then gives the conductivity difference, but no information on the absolute conductivities. Compared with absolute EIT, this method is more robust to noise and less sensitive to poor electrode placement. However, without the correct modeling, the forward model might not be able to fit the data by adjusting the conductivity [25], and nonlinear iterative methods might fail to converge.

Another method, similar to EIT, called Electrical Capacitance Tomography (ECT) has a very similar inverse problem. However, in the case of ECT, the inverse problem aims to reconstruct both the real and imaginary part of the electrical impedance, hence the electrical permittivity. Though, the application presented here and the low frequency of the data acquisition system makes permittivity measured or reconstructed to be insignificant when compared with the real part of impedance. Therefore, it was decided to focus on the real part of the electrical impedance.

### Artificial neural networks

ANNs are powerful AI algorithms for finding an optimal solution to a nonlinear problem.

In this paper a specific category of ANN is used: the radial basis function (RBF) network [26]. An RBF is a function that has been built into a distance criterion with respect to a center. RBF ANNs are a category of ANNs in which RBFs are used as the neurons’ activation functions. In the RBF networks used in this paper, all of the input neurons are interconnected to all of the neurons present in a hidden layer, which automatically generates a larger number of weights and biases as the neurons become larger.

The mathematical model of the RBF is given by (5):

$$y\;=\;exp(-{\left(\frac{∥x-t∥}{\sigma }\right)}^{2})$$ (5)

where *x* is the input data sent to the neuron, *t* the center of the RBF function, and *σ* the spread constant. Although the spread constant plays an important role in the quality of the images [27], actual training algorithms are not capable of automatically finding the best parameters for the variables within the transfer functions themselves. A common practice is to consider a spread constant of 1 and a center of 0.

Several works on ANNs as a replacement for EIT inverse solvers have been published, and they have produced good results in simulations and phantom experiments. However, very few studies have reported ANN-based inverse solvers for real biomedical applications. To the best of the authors’ knowledge, only linear ANNs have been used to image the lungs during breathing [28]. Progress in this area might well have been limited by the difficulty of efficiently training an ANN for real biomedical applications. In short, an inverse solver based on an ANN gives good results if, and only if, the training data are close enough to the real data. In biomedical applications, it is difficult to consider all of the possible distortions that can occur in practice when modeling the EIT problem and generating the training data from simulations. In image processing, ANNs are often utilized to perform complicated tasks such as classification [29] or object recognition [30]. In this case, it is common practice to use various tools, such as image segmentation and feature extraction, before using the ANN [31]. These additional steps are carried out to reduce the complexity of the problem that the ANN has to solve. It can therefore be said that ANNs are utilized as a post-processing tool in several imaging applications.

The novelty of this study lies in combining the use of a linear EIT inverse solver and an ANN for two-dimensional EIT, in which the ANN is used as a post-processor to eliminate the linear aspects of the resulting image, reduce the smoothness, and therefore obtain a more realistic conductivity distribution.

**Fig 1:**
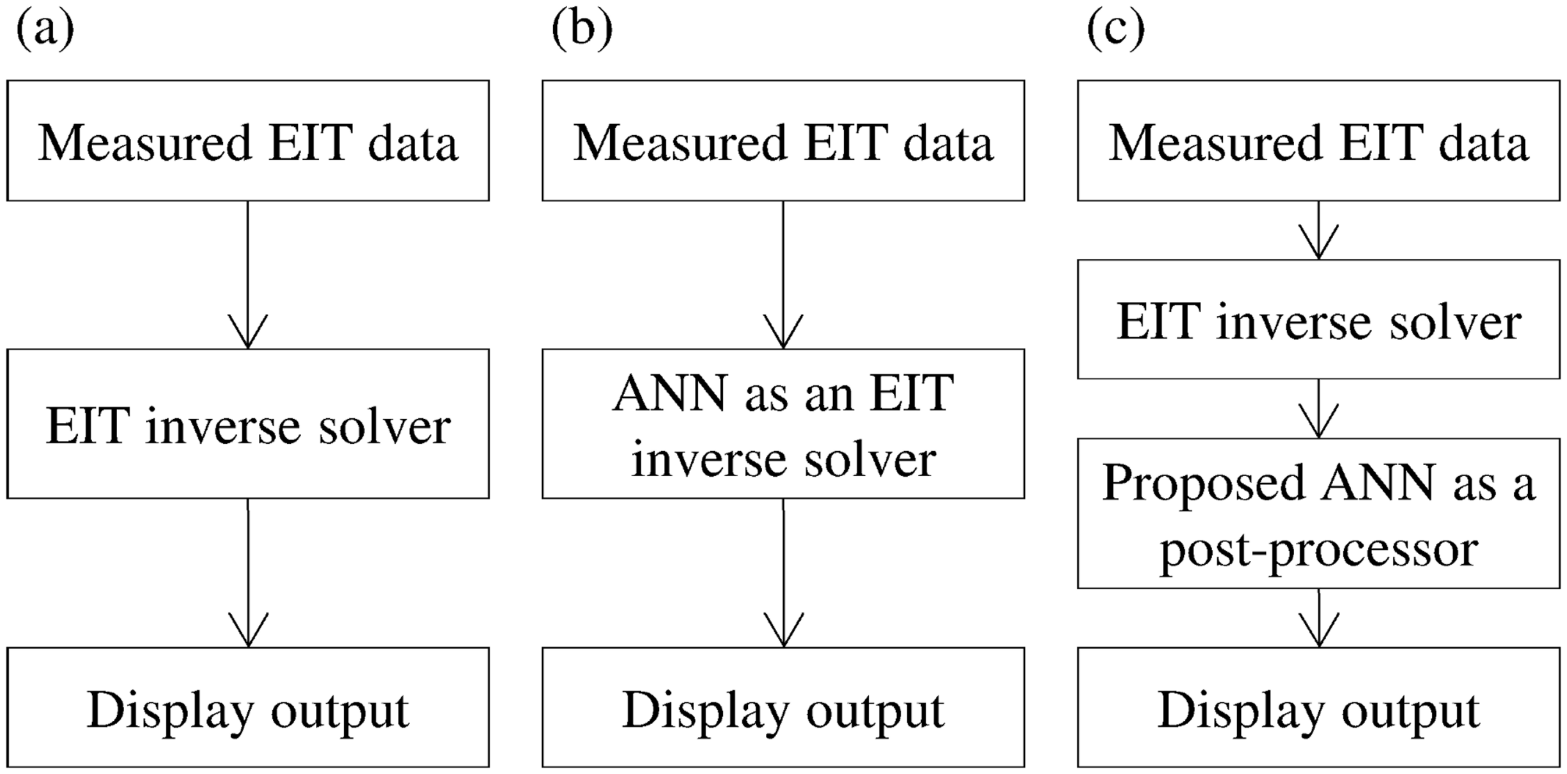
Comparison between existing methods and the proposed method. (a) The general EIT reconstruction method, (b) an ANN used as an EIT inverse solver, highly sensitive to modeling errors and noise, and (c) the proposed reconstruction method, less sensitive to measurement errors. In (c), the input of the ANN is the output of the inverse solver.

Applying the ANN after solving the inverse problem with a linear inverse solver gives greater convergence to the correct output and minimizes the risk of failure. In fact, ANNs, when applied for nonlinear reconstruction, are very sensitive to boundary mismatch and other artifacts in the measured data. Linear algorithms are known to be robust against noisy input data (measured voltages) and inaccurate modeling, and consequently are expected to eliminate the artifacts generated by noise or modeling errors. It then becomes possible to train the ANN without noise and without considering movement in the training data, while still lowering the risk that the ANN will “see” a previously unencountered pattern, and thereby reducing the difficulty of extrapolation [32]. With the proposed method, it becomes easier to generate efficient training data and thus reduce the error. A simple comparison between the existing reconstruction method, based on an ANN as a replacement for an inverse solver, and the proposed method, based on an ANN as a post-processing technique, is shown in Fig 1.

A similar method has been applied for three dimensional EIT image reconstruction [33]. However, in this case, the inverse problem is relatively larger and computationally demanding to solve. As in biomedical applications, especially for monitoring pulmonary activity, the electrodes are usually all connected to the same plane, all the relevant information is available on a 2D plane. Therefore, in this paper, the post-processing method is applied for two-dimensional EIT imaging. Compared to a 3D reconstruction, the method proposed here is applicable in real-time on any modern computer.

## Materials and methods

To verify the aforementioned hypothesis, two-dimensional (2D) EIT simulations and image reconstructions were designed to train the ANNs. As the goal of the ANNs is to improve the output of the linear inverse solver and reintroduce nonlinear variations from the initial guess into the estimated conductivity distribution, their input should be the solution of the inverse problem obtained by the linear solver and their output the original conductivity distribution, used at the starting point of the inverse solver. During the training phase, the output was the original conductivity distribution, simulated by computer. Up to 2000 EIT forward and inverse problems were solved to obtain an efficient dataset for training. While a larger dataset would give higher accuracy, experiments have shown that the quality does not increase much when using more than 2000 samples. Each of the 2000 EIT simulated conductivity distributions contained one or two targets randomly distributed in space, having random conductivity, contrasting with the background conductivity. The electrical properties of the target and the background were designed to match the electrical properties of the saline solution (9 grams of NaCl per liter of water) and the acrilyc (an excellent insulator having a resistivity above 10^15^ ohms.m) present in the phantom, or the electrical properties of physical tissues in case of lungs imaging. For instance, in the case of lungs imaging, the lungs are expected to have an electrical resistivity varying from 700 to 2500 ohms.cm. Then, the simulated conductivity distribution were designed accordingly. Since the electrical conductivity of the lungs is already known to be within a certain range, this assumption seems to be fair. For simulations and phantom experiments, the training data contains targets of different shapes, elliptical, triangular, or rectangular shapes. For in vivo reconstruction, the training images consist of two elliptical targets only. The size of the targets varies from 5 to 40% of the area of the 2D model.

The FE model used for the phantom experiments was a circular tank, divided into 1600 triangular elements, with 16 electrodes located at the boundary. For reconstruction of the lungs, the shape of the FE model was adjusted to resemble the human thorax. This deformation was performed using the Fourier descriptors obtained from computed tomography (CT) images. After acquisition of the voltages from a healthy patient, images were obtained using the EIDORS toolkit [34] and MATLAB’s neural networks toolbox, running under an Intel Core I7 CPU and Ubuntu Linux.

### Data acquisition and inverse solver

Phantom data, as well as lung data, were acquired using the data acquisition system presented in [35]. This system uses pairs of adjacent electrodes for current injection and voltage measurements. For each current injection, 16 voltage readings were measured with the 16 electrodes present at the boundary of the volume conductor. The current source was then moved to the next pair of adjacent electrodes and the measurements were repeated. This process was performed for all 16 pairs, generating 256 measurements for image reconstruction. Although only 104 of these measurements were independent, it is common practice to keep all of the measurements to reduce the influence of noise [36].

For both the one-step GN and PDIPM solvers, the Jacobian matrix was obtained using the adjoint method, and the prior probability function was introduced with the Newton one-step error reconstructor (NOSER) reconstruction method [37]. The problem was solved by iterative methods based on five iterations.

#### Noise estimation and modeling

The presence of white Gaussian noise (WGN) was considered during the phantom experiments. Although most of the noise was cancelled by using a tenth-order bandpass filter centered on the frequency of the injected current, some noise was still present in the acquired data. To reduce the difficulty of extrapolation for the ANNs post-training, it was of interest to train them with noisy data similar to those acquired from the phantom.

The amount of noise was not fixed and strongly depended on the physical distance between the current source and the electrodes used for measurement. Hence, the noise was estimated independently for each measurement. Current injection consisted of a sinusoidal waveform at a frequency of 100 kHz. During each sine wave, 20 voltages were measured by each pair of electrodes. For EIT reconstruction, these data were filtered and only the highest peak was retained. The noise was estimated by comparing the measured data with a simulated sine wave. In estimating the amount of noise in the measurements before filtering, one can assume the noise to be WGN. Noise was thus estimated according to (6):

$$SNR\;(dB)\;=\;10*\text{log}(\frac{mean({signal}^{2})}{mean({residual\;noise}^{2})})$$ (6)

**Fig 2:**
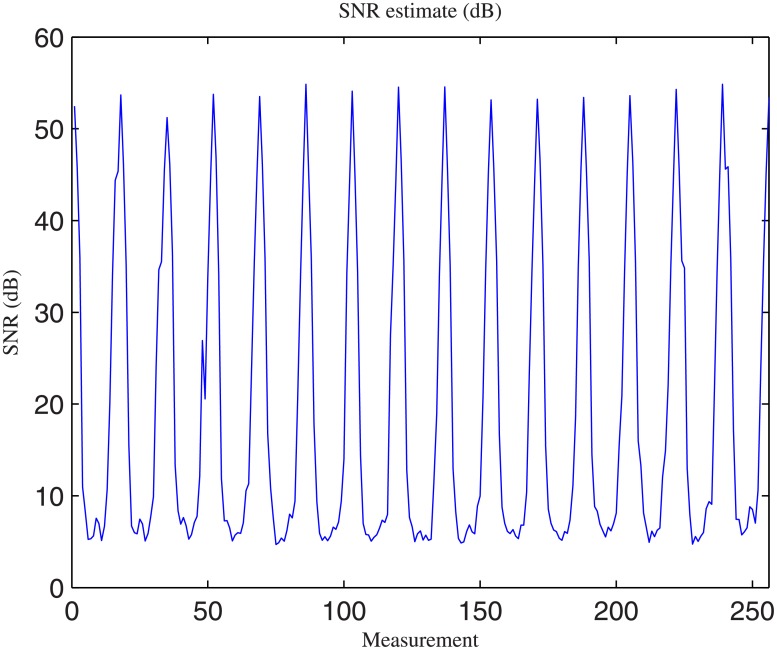
Noise estimate obtained from phantom data. Noise was evaluated on each of the 256 measurements before filtering. For each current injection, voltages were measured by each pair of adjacent electrodes.

Finally, the signal-to-noise ratio (SNR) for each of the 256 measurements was estimated over 500 frames, and the average signal was considered the expected signal. Fig 2 shows the estimate of the noise for each of the 256 measurements. This estimate was obtained from the phantom measurements from the EIT data acquisition system described in [35]. When the measured signal was spatially close to the injected current, the estimated SNR was above 50 dB. However, when measured at the opposite side from the phantom, the SNR was sometimes less than 10 dB. By assuming WGN before filtering, it was possible to reproduce the noise by adding a source of WGN to the simulated sine waves. Then, the presence of a filter was simulated to generate a noise model that was close to the noise present in the real phantom experiments.

Two different noise models were generated, one for the phantom data and one for the lung data. For the phantom data, one can assume an additive WGN, while for the lung data one has to consider physiological phenomena inducing non-Gaussian noise. For the lung data, the noise level was evaluated with the same EIT hardware used for the phantom experiments, using data obtained from a healthy volunteer. During this data acquisition, a spirometer was used to monitor the breathing state. To measure the amount of noise present in the lungs data, all the filters were removed and the raw data (sine waves) were collected. By removing all the filters, we could analyze the raw signal measured directly from the electrodes, This signal is expected to contain some WGN from the common mode noise, mixed with noise from physiological processes, such as breathing and heart beat. The use the spirometer combined with EIT is a simple method to easily distinguish the inspiration and expiration phases. The noise was measured and averaged over a hundred samples at the end of inspiration and at the end of expiration phases. Finally, to train the ANNs after solving the forward problem on simulation data, a similar noise was added artificially on the raw signal, which was then filtered with a filter similar to the one actually used to acquire data. By doing this, it is expected the training data will contain a similar noise to the actual biomedical measurements.

Note, however, that for ethical reasons, the images of lungs presented in this paper were obtained from a clinical environment where different hardware was used. Although the physiological phenomena were expected to be similar, it could not be guaranteed that the noise model would be identical to one measured on a different hardware system.

#### Boundary of lungs

For experiments involving real lungs, the presence of unknown and variable contours is an additional challenge. Although the shape of the thorax can be approximated using Fourier descriptors obtained from reference CT images, the actual shape not only is different for each patient, but also changes with time. For example, the shape of the thorax slightly varies during inspiration and expiration phases, and consequently, the FE model used for image reconstruction for time-difference EIT can only approximate the exact shape.

**Fig 3:**
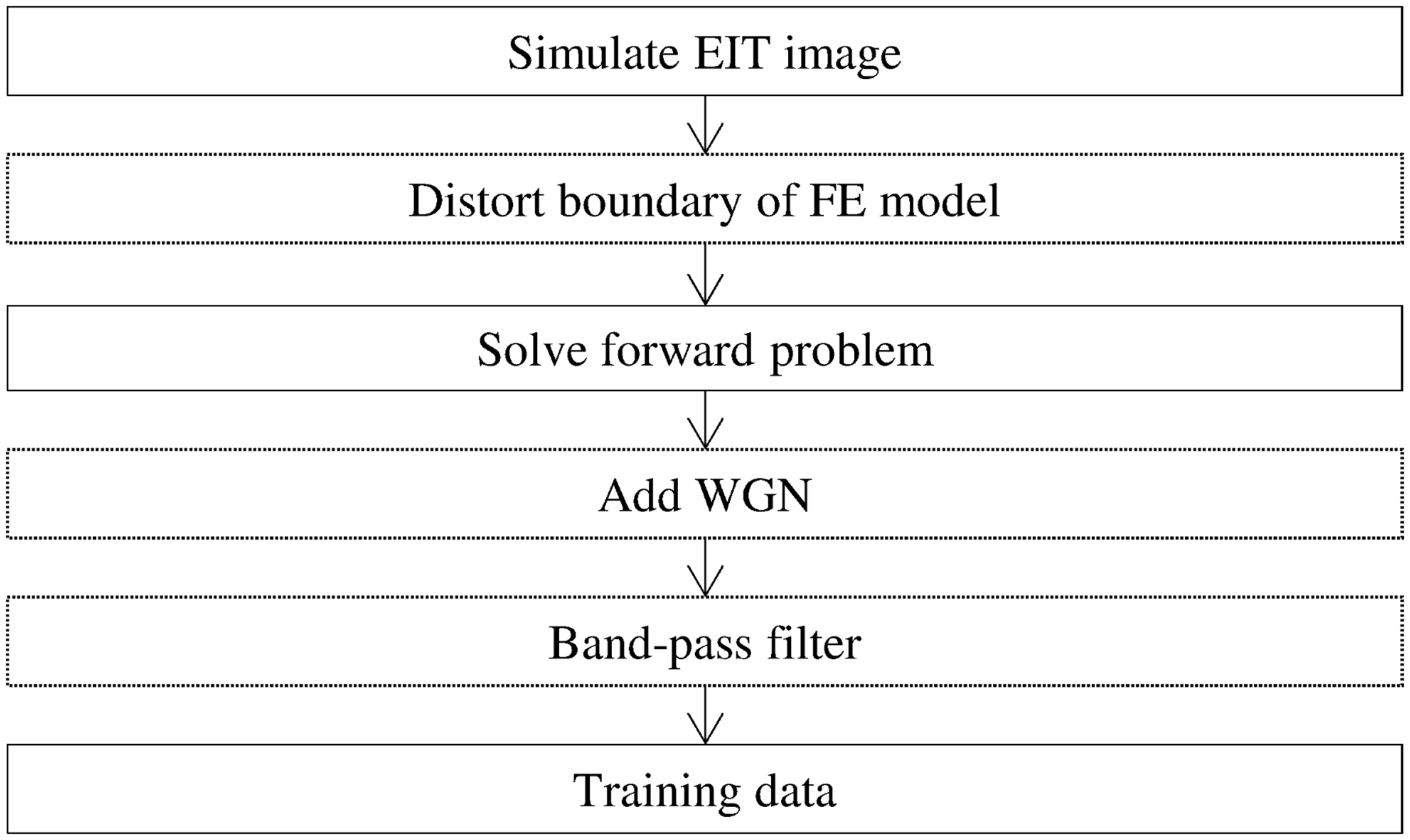
Flowchart of the method used to generate voltages to train the ANNs. Text within dotted lines represents optional steps. For the proposed post-processing, the inverse problem was solved using an undistorted FE model after generating the voltages and before training the ANN.

To anticipate the risk of inexact modeling, the ANNs were trained to consider different boundaries. Different conductivity distributions were simulated during the training phase, and their corresponding measurements at the electrodes were obtained by simulating the EIT forward problem. To train the ANNs for post-processing application, the inverse problems were solved as well. While the inverse problems were always solved using the same FE model, the models used for solving the forward problems were varied. For each simulated EIT image, the Fourier coefficients representing the shape of the thorax were slightly adjusted to generate a different shape before solving the forward problem and obtaining the corresponding voltages at the electrodes. Fig 3 illustrates the method for solving the forward problems to train the ANNs. This method is known to give the ANNs a higher tolerance to the possible mismatch between the boundary of the FE model and the boundary of the actual volume conductor [28].

By distorting the shape of the FE model before solving the EIT forward problems, the resulting training data forced the ANNs to learn to perform reconstruction from distorted thorax shapes and inexact modeling. Clinical EIT measurements, taken from the thorax of a healthy patient, were used to demonstrate the applicability of the proposed method for data from real lungs. Those measurements were carried out with a commercial EIT system, consisting of both software and hardware, which was used at 100 kHz for data acquisition. The signal was then filtered to reduce the possible interferences from the heart and from breathing.

### Artificial neural networks

**Fig 4:**
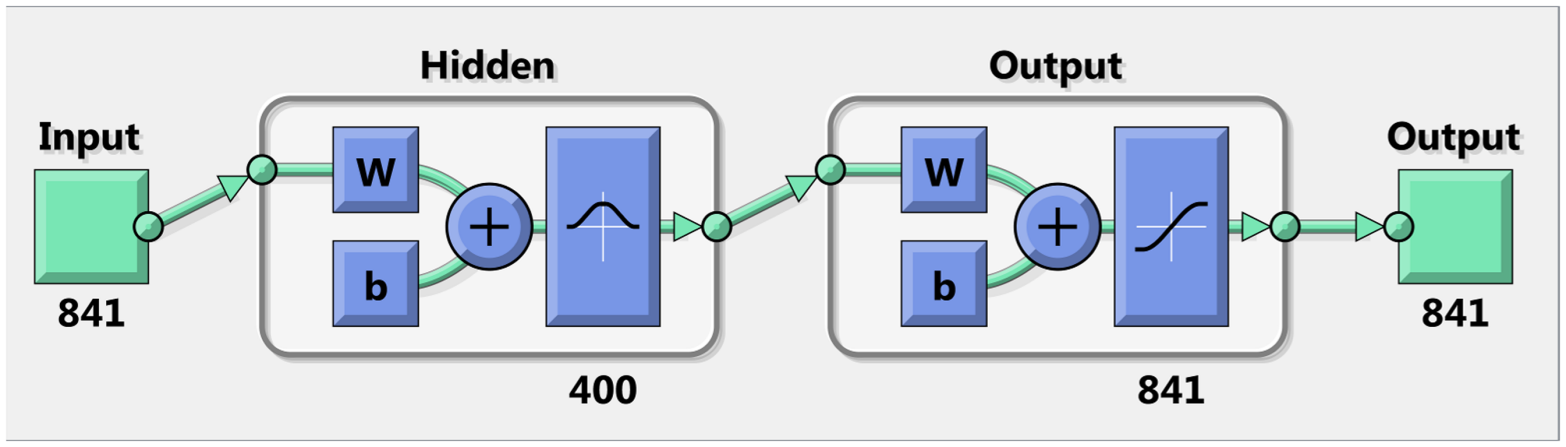
Configuration of the ANN used as a post-processor. This configuration is used in the flowchart shown in Fig 1(c).

The ANNs were used to improve the image quality after reconstruction, the images having been obtained using the one-step GN method. One-step GN is known to be resistant to noisy data and inaccurate modeling. It consists of a simple matrix product, which can be very rapidly computed if the matrix is already known. The high robustness to noise and fast reconstruction makes the linear one-step GN a suitable algorithm for EIT image reconstruction before applying the ANN for post-processing. An RBF ANN, used as a post-processor, is shown in Fig 4. The FE model consists of 841 nodes, and 400 neurons are present in the hidden layer.

To demonstrate the viability of the proposed post-processing with an ANN, the resulting images were compared with images obtained by the iterative PDIPM after five iterations, and with the reconstructions obtained by an ANN used as a complete replacement for the inverse solver. In the latter case, the ANN had the same configuration as that used for post-processing, and was trained with the same generated images, the only difference being the input dataset. In other words, 2000 conductivity distributions (or images) were generated. Starting from those conductivity distributions, for an ANN used as an inverse solver, only the forward problem was solved and the resulting voltages were used as input data. For the proposed post-processing, both the forward and inverse problems were solved, as the input of the ANN is the solution to the inverse problem.

### Error quantification

Various error functions have been proposed for medical imaging applications. Several normalized error definitions are also available [38]. Among these normalized definitions, position error (PE), resolution (RES), and shape deformation (SD) errors are of significant interest and were calculated here.

**Fig 5:**
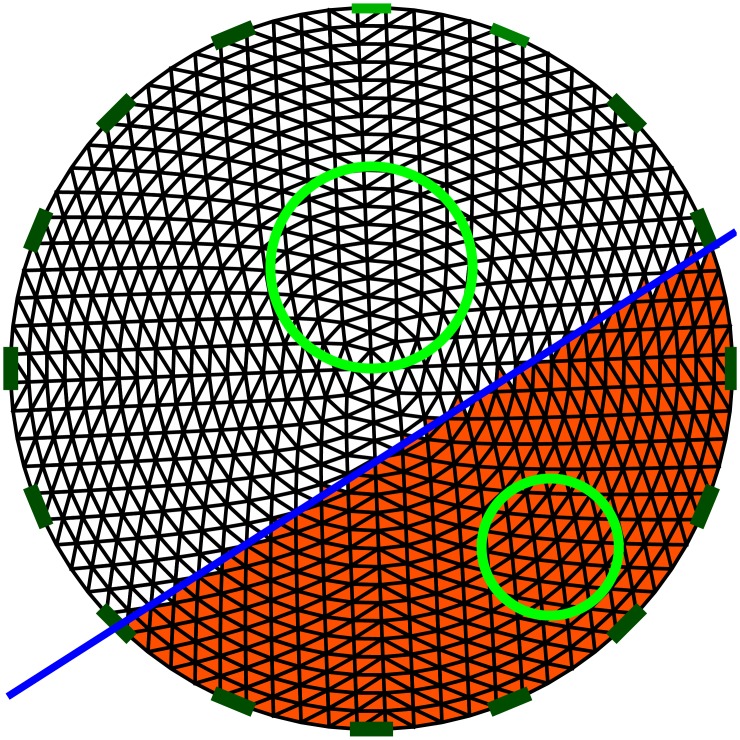
In the case of two targets, the resulting image is split into two images. Splitting is performed by drawing a line between the two targets. The line is equidistant to the centers of gravity of the two targets. The targets are shown by a green circle, and the FE model is split by the blue line.

PE estimates the distance between the center of gravity of the target and the center of gravity of the reconstructed object over the diameter of the tank. To do so, the resulting conductivity distribution is represented by a 32 × 32 pixel image, and is thresholded so that all of the pixel data within 25% of the maximal conductivity or resistivity are considered as part of the reconstructed object. In the case of multi-target images, the resulting image is divided into two parts by a line between the centers of gravity of the two targets, so that a different error is obtained for each target. Fig 5 illustrates how the separation is performed. The PE is then the ratio of the distance between the actual location and the estimated location to the diameter of the water tank, so that the maximum PE is 100%.

RES estimates the radius ratio of the reconstructed target as a fraction of the medium. It can be understood as a ‘size-error’ and should not be confused with the definition of resolution commonly used in image processing. In the case of a circular target, the expected RES value is equivalent to the radius of the target object. For non-circular targets, the expected RES value increases with the area or volume of the target. Typically, when the target object is large, a large RES is expected, and vice versa. To define the error, the difference between the RES obtained for the initial and reconstructed images, |ΔRES|, is used. Linear solvers, such as one-step GN, have a tendency to output larger objects, and hence larger RES errors, especially when the available a priori information included in the prior is limited.

Reconstruction algorithms sometimes generate a target with a similar area to the original object, but containing large artifacts as a result of smoothness, and differing from the expected shape. In such cases, SD defines the error as the fraction of the reconstructed target that does not fit in the expected position.

This paper now presents the results obtained with difference EIT, and focuses on the location, shape, and visibility of the targets rather than estimating the true conductivity.

## Results

### Simulation

EIT simulations were conducted to validate the robustness of the proposed method to errors in boundary modeling. In practical medical applications of EIT, the boundary of the volume conductor varies with time and cannot be exactly known at the time of the experiment. It is thus important for the proposed method to have a strong robustness to such distortions.

**Fig 6:**
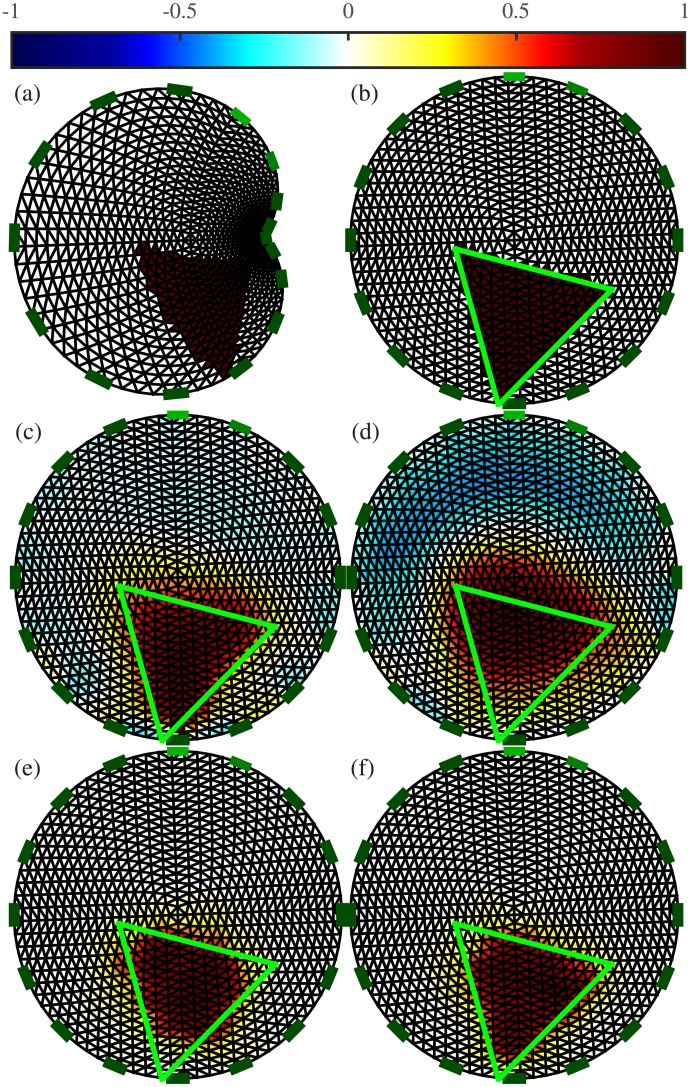
EIT image reconstruction from simulated data. The forward problems were solved with distorted models. (a) Simulated distorted FE model used to solve the EIT forward problem, (b) the projection into the circular undistorted FE model. Reconstructions were obtained with (c) one-step GN, (d) PDIPM, (e) an ANN used as a complete inverse solver, and (f) the proposed post-processing with an ANN. The contour of the target (projected into the circular model) is shown in green. On top, the normalized resistivity distribution.

Different conductivity distributions, each showing a target of a random shape, were generated in a circular mesh. The conformal deformations were then applied to the FE models, and the forward problems were solved with these different models. At this stage, the resulting voltages were available to train an ANN as an inverse solver. However, for the proposed post-processing application, the inverse problems had to be solved before training the ANN. Assuming that the actual contour of the FE models from which the voltages were obtained was unknown, the inverse problems were solved by considering a circular mesh. The resulting conductivity distributions are shown in Fig 6.

Using EIT to image a triangular shape, as in Fig 6, is complicated by the large amount of smoothness generated by the reconstruction algorithms. This smoothness obscures the boundary of the reconstructed inclusion, potentially resulting in a near-circular rather than triangular image. Fig 6(a) shows that even when the target is located at the expected position, the smoothness in the resulting image limits the performance of linear methods. Although nonlinear algorithms may perform better, they are highly sensitive to inaccurately modeled boundaries as well as inexact electrode positions, which are unavoidable sources of error in time-difference EIT. Fig 6(d) shows that the nonlinear iterative PDIPM has difficulty in accurately imaging the target and generates large artifacts.

As shown in Fig 6(e) and 6(f), the ANNs are trained on datasets containing modeling errors (introduced by applying random complex conformal deformations, as shown in Fig 6(a)) when solving the forward problem. In principle, therefore, they should be capable of EIT reconstruction under those circumstances. Although both methods are capable of EIT reconstruction, the ANN as an inverse solver does not give the expected triangular shape.

Fig 6(f), in contrast, shows that the proposed method not only estimates the conductivity distribution with satisfactory accuracy, but images a triangular target with limited smoothness and some distortion.

For each of the four reconstruction methods, the resulting PE and |ΔRES| errors are reported in Table 1. The reconstruction obtained with the PDIPM does not accurately estimate either the position or the size of the target, resulting in the highest values of PE, |ΔRES|, and SD. The two methods using ANNs perform significantly better than the one-step GN method and the PDIPM. Using an ANN as an inverse solver gives a PE of 8.80%, a |ΔRES| of 4.01%, and an SD of 22.90%. Finally, the proposed post-processing method gives a PE of 1.66%, meaning that the Euclidean distance between the centers of gravity of the reconstructed and initial targets is only about 1.40% of the diameter of the mesh. The |ΔRES| error below 4% indicates that the target and reconstructed target have comparable sizes. In addition, the low SD of 16.18% means that the shape of the reconstructed target is less distorted with the proposed post-processing method than with the three other methods discussed in this paper.

**Table 1:** PE and |ΔRES| errors obtained for EIT simulations with different methods.

Method	PE (%)	|ΔRES| (%)	SD (%)
One-step GN	12.44%	11.07%	19.63%
PDIPM	20.81%	19.91%	25.77%
ANN as inverse solver	8.80%	4.01%	22.90%
One-step GN + ANN	1.66%	3.70%	16.18%

Corresponding images can be seen in Fig 6. The proposed post-processing (one-step GN + ANN) gives the lowest errors.

As a validation, 2000 additional images, different from the training data, were generated. On those 2000 images, the location, shape, and conductivity of the target, as well as the model distortions, were random. The background conductivity and the amount of noise injected in those data remained constant. Then, the average errors were computed and are reported in Table 2. The mean errors are close to those in Table 1. In both cases, the proposed method gives the lowest mean errors, as well as the lowest standard deviation for PE and |ΔRES|. A low standard deviation is an indication of stability. The low error and high stability of the proposed method over the reconstruction of 2000 different random images shows the proposed method is capable to deal efficiently with the reconstruction of different targets and different shapes.

**Table 2:** Mean errors and standard deviation obtained from 2000 simulations.

Mean ± Standard deviation	PE (%)	|ΔRES| (%)	SD (%)
One-step GN	11.93±4.37%	14.61±4.98%	18.36±9.22%
PDIPM	21.32±8.24%	23.77±7.11%	26.32±9.18%
ANN as inverse solver	7.86±6.24%	4.84±4.43%	21.45±9.25%
One-step GN + ANN	2.14±1.13%	4.42±3.02%	17.01±9.38%

### Phantom experiments

After validating the proposed idea with EIT simulations, real phantom experiments were conducted. A circular model composed of 16 equally spaced electrodes was filled with ionized water and used to acquire data. Two electrical insulators, made of acrylic, were placed in this phantom as a target object. The ionized water (9 grams of NaCl per liter) has an electrical conductivity of 0.9S/m in at 20 degrees C, while the electrical insulator is assumed to have a very low conductivity, close to zero. Current injections and voltage measurements were carried out with and without the presence of the target (time-difference EIT). Although the common-mode noise was removed by performing differential measurements, the data acquired from this phantom still contained noise of the WGN type, which might have strongly affected the quality of the EIT image reconstructions.

First, reconstructions were carried out using ANNs trained on noise-free datasets. Then, additional reconstructions were carried out using ANNs trained with the inclusion of noise in the simulated voltages.

**Fig 7:**
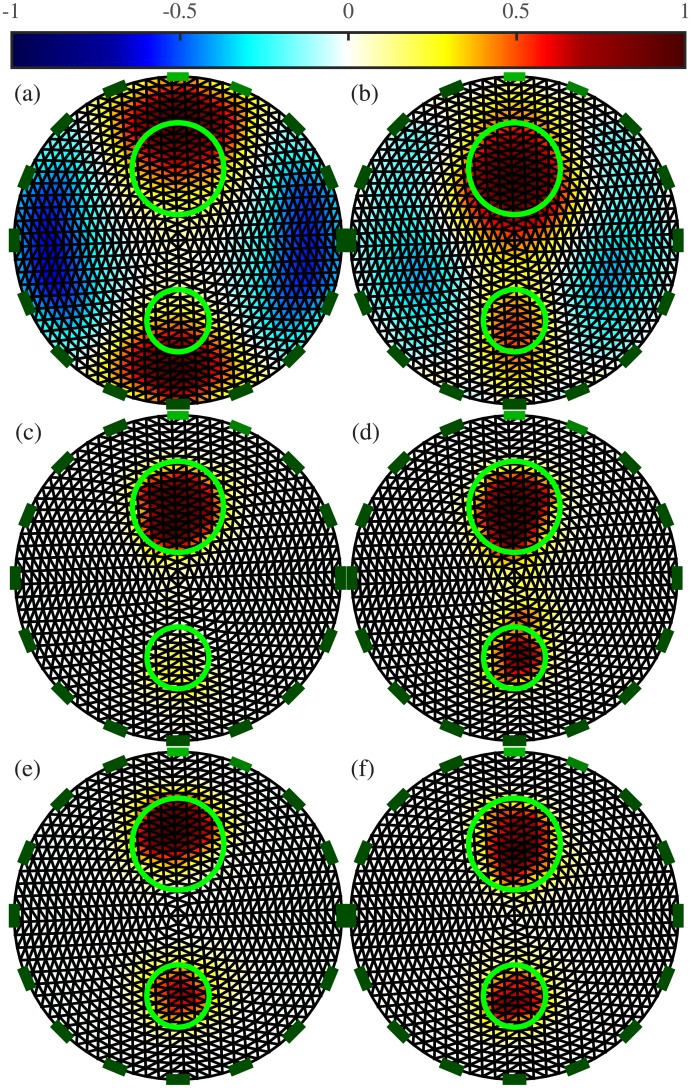
EIT reconstructions from phantom data with different algorithms and the proposed method. (a) One-step GN, (b) PDIPM after five iterations, (c) ANN as an inverse solver and (d) as the proposed post-processor (ANNs in (c) and (d) are trained without noise), (e) ANN as an inverse solver and (f) as the proposed post-processor (ANNs in (e) and (f) are trained with noise). The contours of the original targets are shown in green. The normalized resistivity distribution are shown on top.

Fig 7 shows the resulting EIT images obtained with an ANN as a replacement for the inverse solver, and with the proposed ANN as a post-processing step, each trained both with and without consideration of noise. Images obtained with the one-step GN and PDIPM solvers are shown for comparison. For each image, the errors are given in Table 3. As the data may contain a certain amount of noise, the images obtained with these methods may contain large distortions, and the reconstructed objects may appear significantly smaller or larger than the target. The linear one-step GN generates large smoothness in the reconstructed conductivity distribution (Fig 7(a)), which leads to large artifacts and higher errors. In Fig 7(b), the iterative PDIPM solver gives two targets located around the expected location, but although the targets appear more “blocky” than with the one-step GN method, some smoothness remains visible and the two targets seem to be slightly interconnected.

**Table 3:** PE, |ΔRES|, and SD errors obtained from phantom measurements with different methods.

Method	Target 1 PE	Target 2 PE	|ΔRES|	SD
One-step GN	20.88%	23.63%	16.90%	64.40%
PDIPM	7.76%	3.93%	14.46%	49.40%
ANN (Training: no noise)	3.05%	8.79%	6.86%	16.31%
One-step GN + ANN (training: no noise)	2.48%	6.87%	3.93%	8.53%
ANN (training: noise)	7.11%	2.35%	3.28%	8.95%
One-step GN + ANN (training: noise)	1.47%	2.06%	1.35%	3.28%

Corresponding images are shown in Fig 7. Top: the first (larger) target; bottom: the second (smaller) target.

In Fig 7(c) and 7(d), the ANNs were trained without noisy data, potentially limiting their ability to efficiently process the noisy data obtained from the phantom experiments. It can be seen that both methods are able to reproduce the targets near the expected location. However, the ANN used as an inverse solver gives two targets with different conductivities, and the smaller target seems to have an electrical conductivity close to that of the background. With the proposed post-processing method, the two targets, both made of acrylic, appear to have similar electrical properties. In addition, the location of the targets matches the expected location, shown by the green circles in Fig 7(d). These two observations show that the proposed reconstruction method offers large robustness against noise, compared with the other nonlinear methods.

As stated above, the ANNs were applied again to the phantom data after retraining on datasets containing noise. If an ANN is trained with an appropriate noise model, similar to the noise model present in the phantom data, the resulting ANN-based methods should be capable of more accurate image reconstruction. In Fig 7(e), the ANN is used as an inverse solver. The resulting image shows the presence of two targets having similar electrical conductivity, as expected. Although one target appears to be shifted towards the outer boundary, the targets are close to the expected locations. In Fig 7(f), both targets are located within the green circles. This indicates that when the ANN was used as a post-processor, the targets were reconstructed at the expected location, implying a low PE.

The PE, |ΔRES|, and SD errors are given in Table 3. The linear one-step GN solver, which makes large assumptions on the conductivity distribution, always gives the highest errors. The iterative PDIPM gives lower errors than the one-step GN solver, but still outputs large |ΔRES| and SD errors: 14.46% and 49.40%, respectively. These errors reflect the smoothness and artifacts present in Fig 7(b). Using an ANN as an inverse solver gives low errors when a noisy model is available for training the ANN. When that is not the case, using this method on phantom data generates large artifacts, which in turn generates large errors. One of the two targets is almost invisible in Fig 7(c), resulting in high |ΔRES| and SD errors. In contrast, the proposed post-processing method can be used for phantom data even when no prior noisy model is available for training. In such cases, the proposed method gives |ΔRES| and SD errors similar to those obtained with the ANN used as an inverse solver after training with noisy data similar to the phantom data. If knowledge of the noise model is available before training the ANN, this model can be used to train the ANN as either an inverse solver or a post-processor. This improves the performance of the ANN. Using an ANN as a post-processor after training with noisy data always gives the lowest PE, |ΔRES|, and SD values. This observation testifies to the quality of the proposed method.

The ability to cope with noise without training the ANN on noisy data gives significant practical advantages to the proposed post-processing method, by allowing a simplified generation of training data. Estimating noise and other sources of error in EIT can be relatively laborious in terms of both analysis and modeling for each different EIT hardware model, and assuming an absence of errors facilitates the generation of training data.

### Imaging the lungs

**Fig 8:**
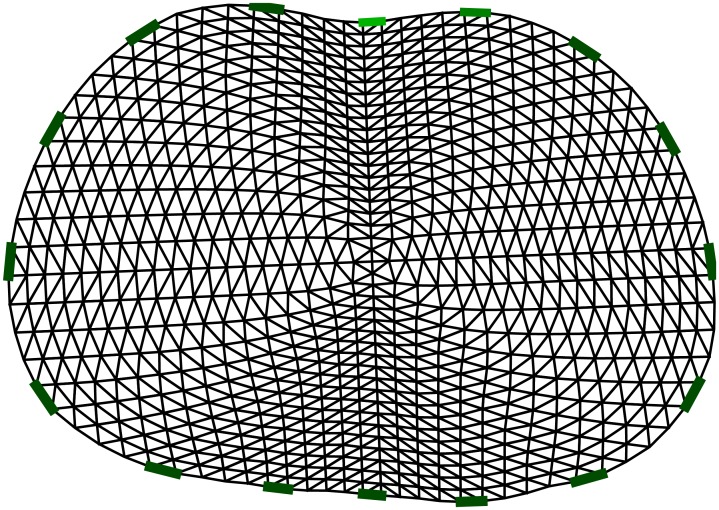
The FE model used for reconstruction of lungs data.

Imaging the lungs is a much more complicated problem than imaging the conductivity distribution within a phantom experiment. In addition to the presence of noise, the exact shape of the thorax, as well as the exact position of the electrodes around the boundary, can only be roughly estimated. Furthermore, the shape of the thorax, and consequently the position of the electrodes, differs between inspiration and expiration. Following manual segmentation of images obtained from CT scans, an average shape of the thorax was estimated. Later on, by using Fourier coefficients, it was possible to obtain a FE model of a shape similar to the thorax shape. The FE model used for imaging the lungs in this paper is shown in Fig 8.

In this study, data were collected over one complete breathing cycle. EIT measurements of the lungs are usually carried out on a single plane, and therefore it was decided to perform a 2D reconstruction. A 2D EIT model was utilized to solve the inverse problem. The result was then fed to the ANN for post-processing. However, a 3D model would also have been applicable [39]. Time-difference EIT was used to image the breathing and visualize the conductivity difference between empty lungs and lungs filled with air. The ANN was trained in two ways. The presence of noise and imperfections in the modeling of the thorax was considered in the first training procedure, and omitted during the second training procedure. To evaluate the proposed method, images were reconstructed from public data, collected at a frame rate of 7Hz, on a healthy patient.

**Fig 9:**
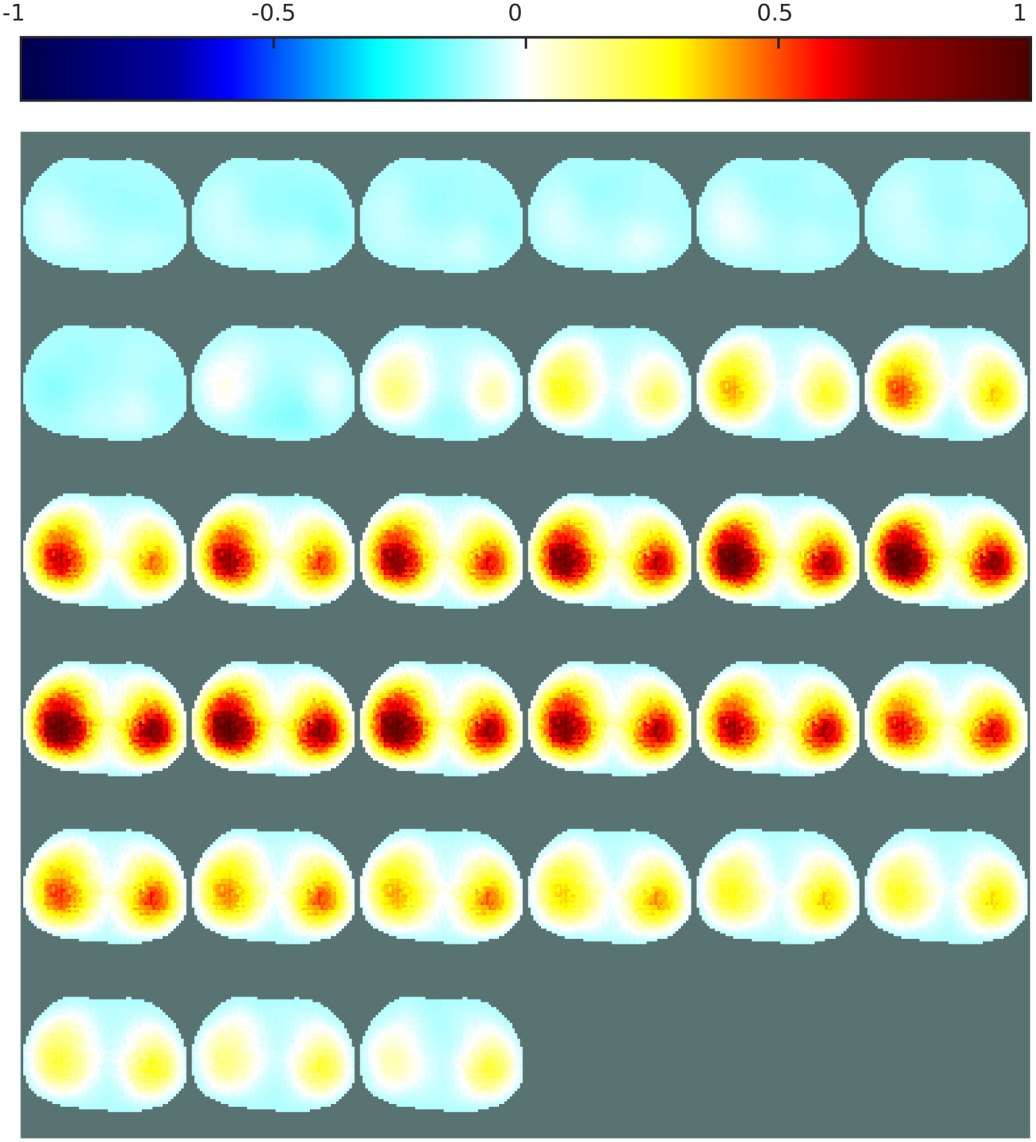
EIT images of the lungs over one breathing cycle, obtained with the one-step GN. Sampling rate: 7Hz.

**Fig 10:**
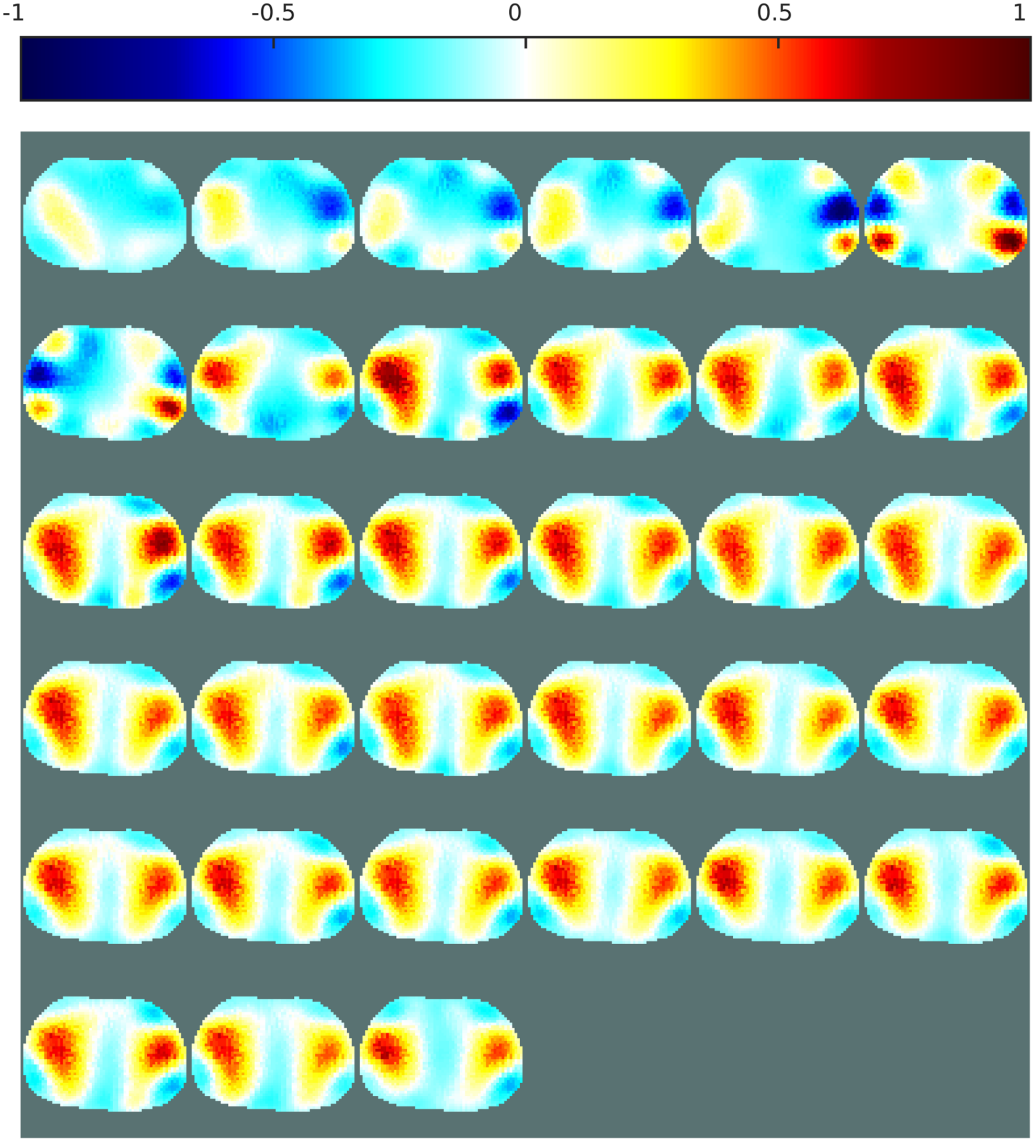
EIT images of the lungs over one breathing cycle, obtained with the PDIPM. Sampling rate: 7Hz.

Figs 9 and 10 show the EIT images obtained with the linear one-step GN and the iterative PDIPM, respectively. Both images display two different regions, corresponding to the variation of electrical conductivity in the lungs during a breathing cycle. In Fig 9, the two lungs are clearly visible but are not of similar size, implying that the breathing of the patient is not symmetrical. As these data were acquired from a healthy patient, this difference of size is more likely to be a limitation of the linear algorithm. In Fig 10, the two lungs are visible, but their shapes are not symmetrical, and the reconstructed images contain artifacts, due to the presence of noise and movement during data acquisition.

**Fig 11:**
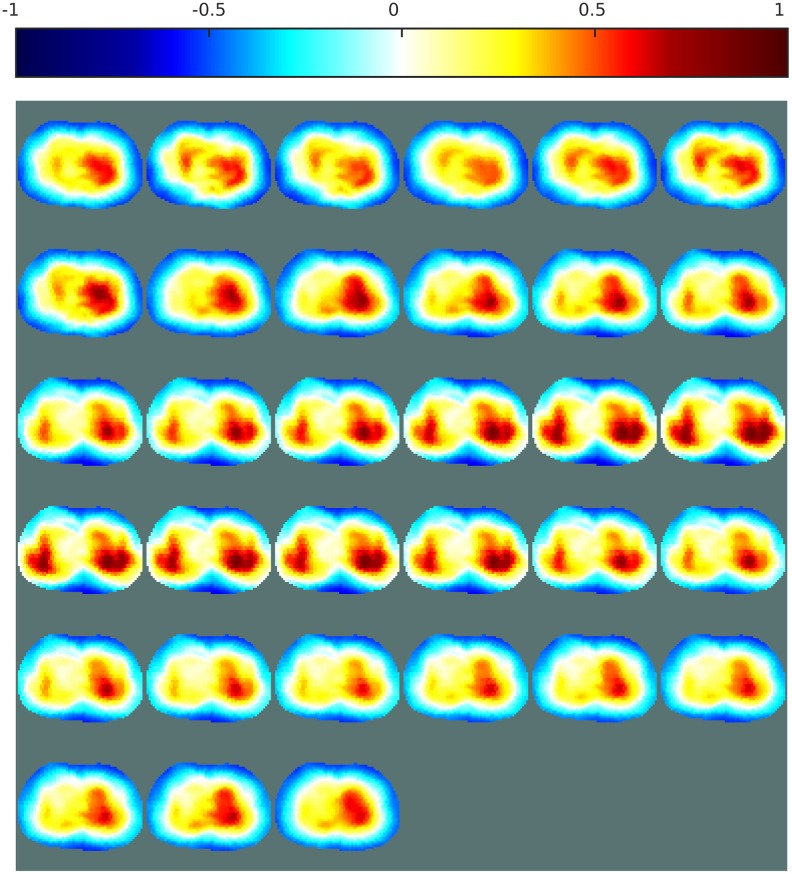
EIT images of the lungs over one breathing cycle, obtained with an ANN. The ANN was used as an inverse solver. The presence of noise and movement was considered during the training. Sampling rate: 7Hz.

**Fig 12:**
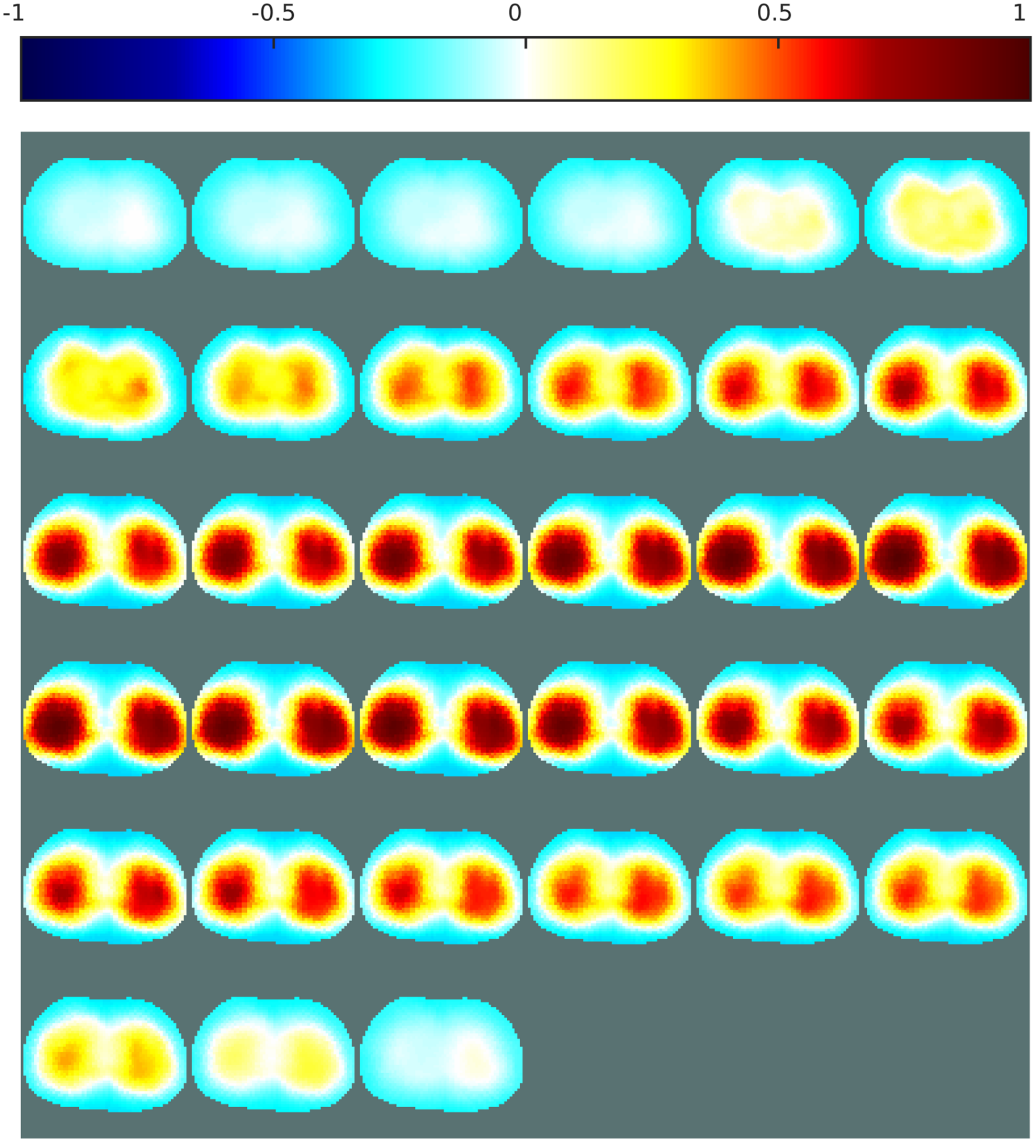
EIT images of the lungs over one breathing cycle, obtained with the post-processing method. The presence of noise and movement was considered during the training. Sampling rate: 7Hz.

Figs 11 and 12 both show reconstructions obtained with ANNs trained on noisy data and a variety of boundary shapes. The noise model here is non-Gaussian and was estimated from real measurements. In Fig 11, the ANN is applied directly to the measured data, while in Fig 12 the ANN is applied to the images obtained with the proposed one-step GN algorithm shown in Fig 9. Applying an ANN directly to the measured data was unstable and sometimes failed to generate a satisfactory image of the lungs. Here, the images generated by this method show two lungs of different sizes and distorted shapes. This relatively poor performance can be attributed to imperfect training of the ANN. As mentioned in the literature, it is crucial to use a complete model that reproduces boundary movement and hardware imperfections in the data acquisition system when training an ANN for use as an EIT inverse solver [40]. For imaging of the lungs, such complete modeling is difficult to achieve. Furthermore, the data were obtained from commercial hardware, and it was impossible to evaluate the noise in these data directly. The noise model used to train the ANNs might have differed from the noise present in these measurements.

When the EIT inverse problem was solved with a linear algorithm before applying the ANN, most of the noise and other errors present in the voltages were eliminated by the linear solver, and the ANN was thus less sensitive to the noise in the measured voltages. Fig 12 shows two clearly distinguishable lungs of similar size. These reconstructions do not contain large artifacts, which confirms that the proposed post-processing method is capable of imaging the lungs during breathing, and therefore that the method tolerates the presence of noise and modeling errors.

The ANNs were then trained without considering possible distortions in the FE models, and without any addition of noise in the measurement.

**Fig 13:**
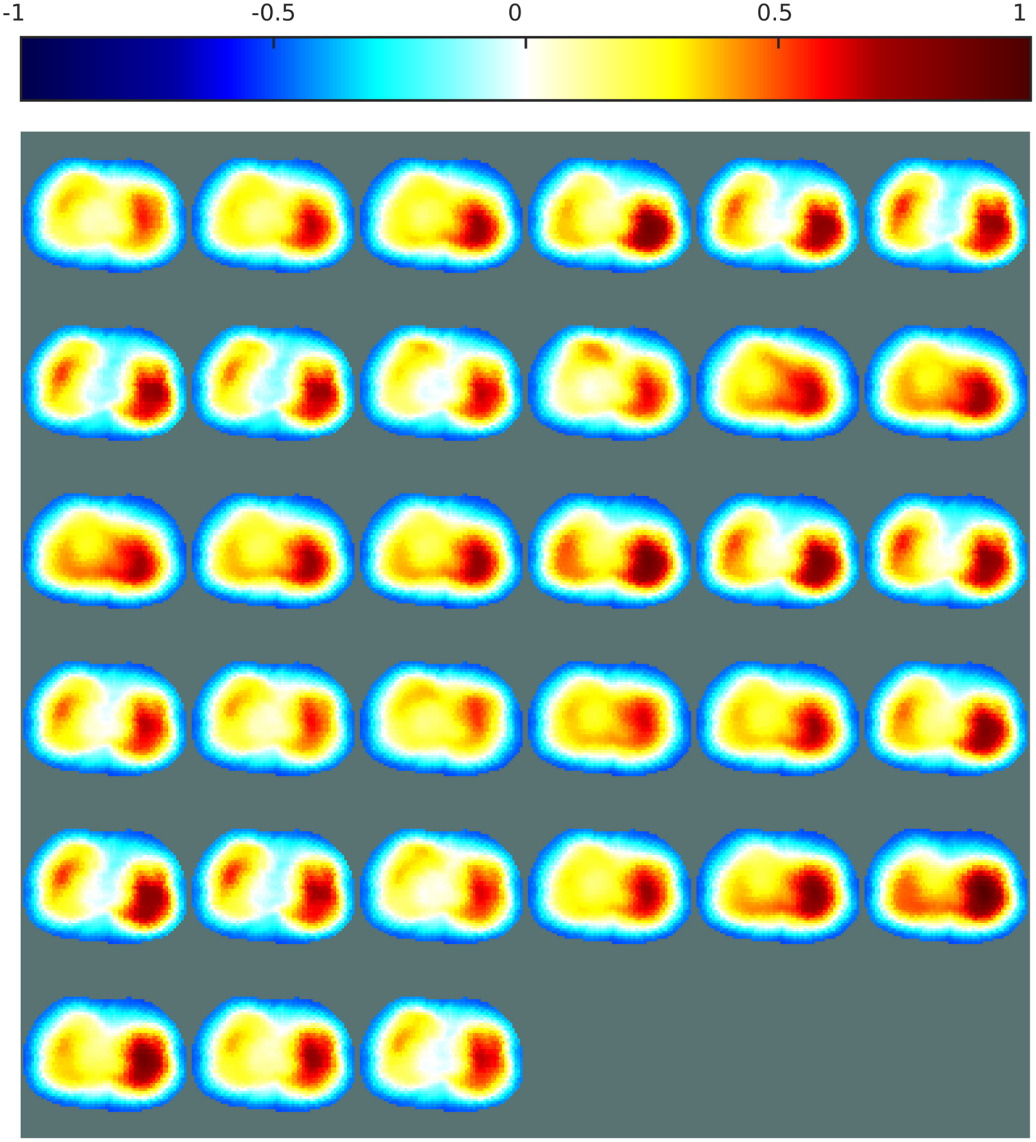
EIT images of the lungs over one breathing cycle, obtained with an ANN. The ANN was used as an inverse solver. The presence of noise and movement was not considered during the training. Sampling rate: 7Hz.

Fig 13 shows the image obtained when the ANN was applied directly to the measured voltages. In this case, only one of the two lungs can be seen and the left part of the images does not show any large conductivity difference, which is not the expected result for a patient breathing normally. This confirms that using an ANN as a replacement for the inverse solver creates high sensitivity to noise and modeling errors, and would thus complicate the generation of training data for biomedical EIT applications, in which noise and movement are important sources of error.

**Fig 14:**
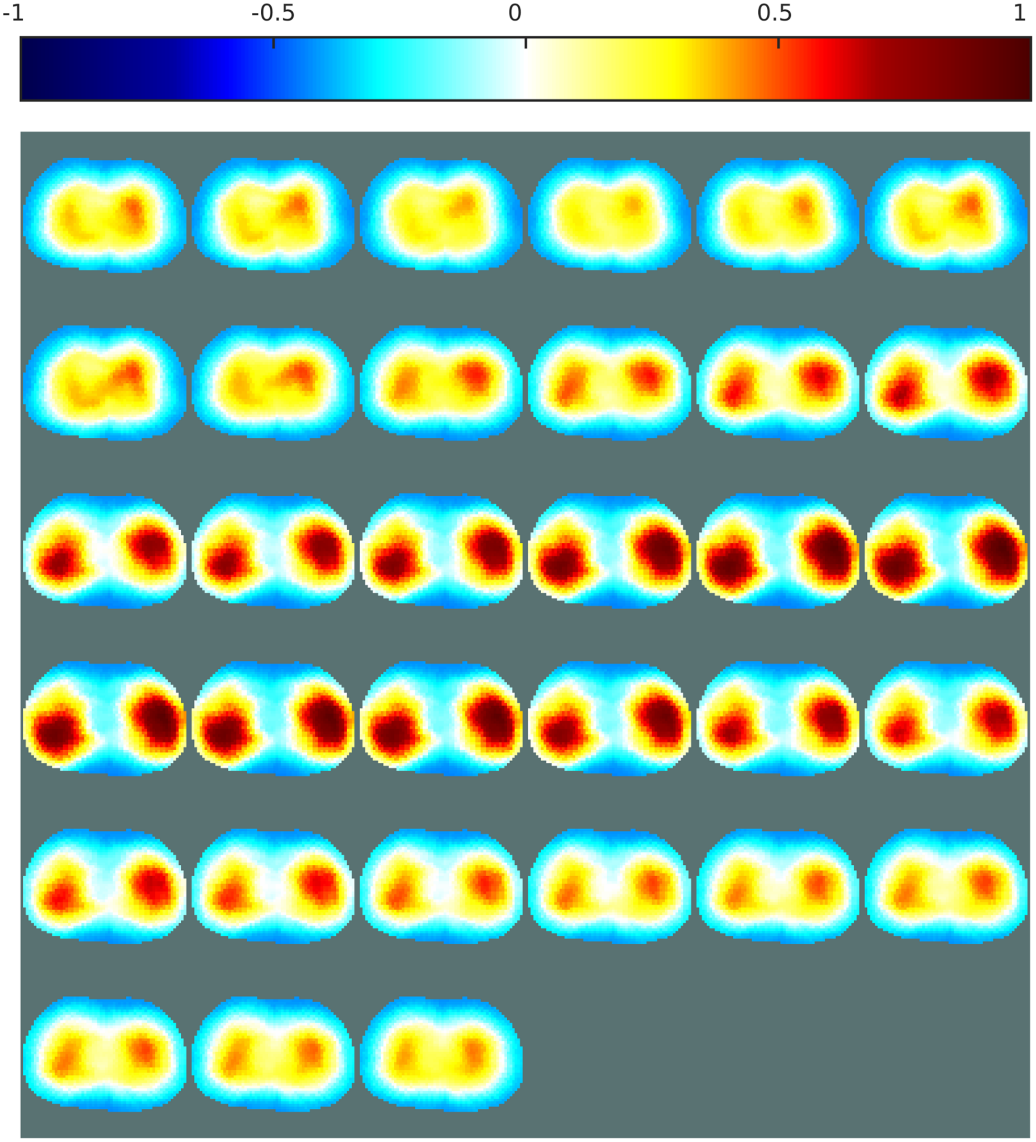
EIT images of the lungs over one breathing cycle, obtained with the post-processing method. The presence of noise and movement was not considered during the training. Sampling rate: 7Hz.

Fig 14 shows the reconstructions obtained when the ANN trained without noise was used as a post-processor after the image was reconstructed from the voltages with the one-step GN algorithm. In this case, the two lungs are still visible and clearly separate, as in the images obtained with the one-step GN. The two lungs appear to be of equal size and electrical conductivity, meaning that they are breathing normally, which is assumed to be accurate as the patient was healthy during data acquisition. Compared with the ANN trained with noisy data, as shown in Fig 12, the artifacts are slightly larger, meaning that training the ANN with noisy data did achieve a slight gain in accuracy and that the noise model was correct.

The results shown in Figs 12 and 14 show that the proposed post-processing technique offers both high-quality imaging and high robustness to noise and modeling errors. The high robustness to modeling errors allows one to obtain a stable and nonlinear EIT reconstruction method based on an ANN and hence develop a stable ANN method for imaging the lungs with higher-quality images than those from linear methods.

Since there is no gold standard for evaluating the quality of EIT images of the lungs, the conclusions presented in this section are rather qualitative. Though, the dataset used to perform the reconstructions of the lungs presented here were acquired at a frame rate of 7Hz during approximately five seconds, which corresponds to the average time of a complete breathing cycle. Therefore the resulting images are expected to show a complete breathing cycle with both the inspiration and expiration phases. The patient being healthy, there should also be some symmetry on the left and right sides of the images, as the two lungs inflate and deflate simultaneously. These aspects are clearly more visible on images obtained with the proposed method than the three other methods used for comparison. Also, the proposed method offers stability between consecutive images. This observation further support the effectiveness of the proposed method over other methods.

### Reconstruction time

For the four methods used in this paper, the EIT reconstruction times were evaluated for the phantom and lung data. The FE models used to obtain the conductivity distributions each consisted of 1600 elements and 841 nodes. The reconstruction times are given in Table 4. It can be seen that the linear one-step GN, which consists of a simple matrix product with a known reconstruction matrix, is capable of approximating a solution in less than 25 ms. The method based on an ANN as an inverse solver requires 55 ms at most and can also be considered as a real-time reconstruction method. The proposed post-processing imaging solution, which combines the one-step GN and an ANN, is by logical necessity slightly slower than the first two methods. However, it is still relatively fast, requiring less than 100 ms. Finally, the iterative PDIPM is the slowest, needing more than 1 s to approximate the conductivity distribution, which is more than 10 times slower than the proposed method. Thus, it cannot be used for real-time imaging.

**Table 4:** Reconstruction time for lung data using the four methods.

Method	one-step GN	PDIPM	ANN	One-step GN + ANN
Phantom (ms)	23	1107	55	95
Lungs (ms)	24	1004	53	91

The proposed post-processing method performs within 100 milliseconds (ms).

Compared to 3D image reconstruction, image reconstruction with the proposed method in a two-dimensional plan is achievable in real-time, Table 5 shows a comparison of reconstruction times between 2D and 3D EIT imaging. Data were acquired at a rate of 7 frames per second, therefore 3D imaging is not suitable for real time reconstruction.

**Table 5:** Reconstruction time for both 2D and 3D imaging of the lungs with the proposed method. The data acquisition rate is fixed at 7Hz or approximately 0.14s.

Method	one-step GN	PDIPM	ANN	One-step GN + ANN
2D (s)	0.02	1.00	0.05	0.09
3D (s)	0.03	5.04	0.13	0.29

## Discussion

This paper proposes a novel method to reconstruct EIT images that combines both linear and nonlinear algorithms. Post-processing the EIT problem with AI-based algorithms allows one to enhance the performance of the classical EIT inverse solver by adding nonlinear behavior into the reconstruction algorithm, thus reproducing the rough boundaries of the tissues even without *a priori* knowledge. Performing these nonlinear operations after a linear reconstruction gives satisfactory results and is compatible with any linear EIT inverse-problem solver.

In the past, attempts were made to solve the inverse problem by applying the ANN directly to the measured voltages, giving satisfactory results but also a very high sensitivity to noise and modeling errors. Such an approach requires training the ANN with noisy data, which must be similar to the noise model present in the hardware system. This depends on *a priori* knowledge of the noise level in each specific system. Therefore, it requires detailed information on the hardware imperfections of the system, as well as sound prior knowledge of the boundary shape and electrode position, especially if this shape is constantly changing, as when monitoring the lungs. In practice, these imperfections are both hardware- and patient-dependent, and may necessitate a new training phase for each monitoring exercise. Presently, although ANN is seen as an efficient EIT image reconstruction method, image reconstruction of the thorax by an ANN as an inverse solver is not yet a reliable clinical solution, mostly because of the difficulties in training the ANN.

Compared with methods based on applying an ANN directly to the measured voltages, the proposed method is less sensitive to the various sources of error that are unavoidable in practical applications, and the generation of the training data can be drastically simplified. This lower sensitivity to noise and modeling errors allows one to image the lungs using a nonlinear ANN with a stable and high-quality output. The risk of overfitting the ANN is significantly reduced, hence opening the way to clinical applications of EIT based on ANNs.

While the noise used in training the ANN to solve the lung problem must be similar to the physiological noise present in human data, this physiological noise varies across patients and hardware systems, and therefore the noise used to train the ANN can only approximate the real physiological noise present in the measured EIT data. Although the noise model used in this study seemed to match that present in the lung data, one cannot guarantee that this will remain the case with a different patient or different hardware. Providing a parameter that indicates the lung ventilation state may help operators to select the most suitable reference signal for each frame. For instance, when the lungs are filled with air, a reference signal measured at the end of the expiration phase would be used, and vice versa.

With the proposed post-processing application, one can use an ANN to enhance the quality of images obtained from a linear inverse solver and train this ANN without considering any sources of error. As linear inverse solvers usually offer strong robustness to such imperfections, applying an ANN after applying the linear inverse solver strongly reduces the risk of the ANN having to process input data that do not resemble the training set. Therefore, when applied as a post-processing tool, the ANN does not encounter previously unseen patterns as often as it would if used as an inverse solver. With the proposed method, noise and distortions during the training of the ANNs do not significantly affect the resulting errors or images.

## Conclusion

Because the EIT problem is severely ill-posed, it is useful to assume a certain level of smoothness in an input image so that the inverse problem can be linearized. However, such a simplification typically results in smoothness in the output images. In this case, it would be desirable to remove this smoothness to obtain more accurate boundary profiles of target objects. The method presented in this paper is able to reduce this smoothness and generate a more accurate conductivity distribution.

To the best of the authors’ knowledge, this paper is the first application of a nonlinear ANN to image the lungs using EIT with the aim of validating the use of ANNs as a post-processing tool for monitoring breathing. In this study, the patient was healthy and breathed normally. Future work will focus on different applications, such as 3D-EIT, and monitoring or imaging the breathing of non-healthy patients using EIT.
